# Neofunctionalisation of the *Sli* gene leads to self-compatibility and facilitates precision breeding in potato

**DOI:** 10.1038/s41467-021-24267-6

**Published:** 2021-07-06

**Authors:** Ernst-Jan Eggers, Ate van der Burgt, Sjaak A. W. van Heusden, Michiel E. de Vries, Richard G. F. Visser, Christian W. B. Bachem, Pim Lindhout

**Affiliations:** 1 Solynta, Dreijenlaan, Wageningen, The Netherlands; 2 Plant Breeding, Wageningen University & Research, Wageningen, The Netherlands

**Keywords:** Plant breeding, Plant genetics, Self incompatability

## Abstract

Genetic gain in potato is hampered by the heterozygous tetraploid genome of cultivated potato. Converting potato into a diploid inbred-line based F1-hybrid crop provides a promising route towards increased genetic gain. The introduction of a dominant S-locus inhibitor (*Sli*) gene into diploid potato germplasm allows efficient generation of self-fertilized seeds and thus the development of potato inbred lines. Little is known about the structure and function of the *Sli* locus. Here we describe the mapping of *Sli* to a 12.6 kb interval on chromosome 12 using a recombinant screen approach. One of two candidate genes present in this interval shows a unique sequence that is exclusively present in self-compatible lines. We describe an expression vector that converts self-incompatible genotypes into self-compatible and a CRISPR-Cas9 vector that converts SC genotypes into SI. The *Sli* gene encodes an F-box protein that is specifically expressed in pollen from self-compatible plants. A 533 bp insertion in the promotor of that gene leads to a gain of function mutation, which overcomes self-pollen rejection.

## Abstract (web summary)

The *S-locus inhibitor* (*Sli*) gene could allow potato breeding by facilitating production of diploid inbred lines. Here the authors show that *Sli* encodes an F-box protein with a promoter insertion enhancing expression in pollen can overcome pollen rejection in the styles of diploid potato.

## Introduction

Potato is the most important non-grain food crop in the world. However, while other food crops such as maize, rice, and wheat have shown a genetic yield gain of 1% per year^1^, genetic gain in potato has been minimal^2^. Currently, most commercially grown potato cultivars are derived from crosses between heterozygous autotetraploid parents. In this breeding system, hundreds of thousands of seedlings are generated and screened in each breeding generation in order to identify those rare individuals that have acceptable characteristics for numerous traits that segregate in the progeny. As there are some fifty traits that are relevant for the value of a commercial potato cultivar, the chance to combine the best alleles controlling these traits using conventional potato breeding is negligible. In addition, targeted introduction of new traits into elite cultivars while maintaining the genetic integrity via backcrossing schemes is impossible without homozygous parental lines. To overcome these problems, several groups have started inbred-line-based diploid potato breeding programs^2–5^. In these programs, genetic gains are achieved via incremental improvements of parental lines by continuously selecting against deleterious alleles during inbreeding and by stacking beneficial alleles in inbred lines through backcrossing schemes^6^. Parental inbred lines are then crossed to produce heterotic F1-hybrid offspring.

In most diploid potato genotypes, inbreeding is severely limited by a gametophytic self-incompatibility (GSI) system that is controlled by the multi-allelic *S*-locus. This *S*-locus encodes style expressed S-RNases that inhibit self-pollen tube growth in the style, preventing self-fertilization^7^. During cross-pollination, pollen-expressed S-locus F-box proteins (SLF) recognize S-RNases and target them to the proteasomal degradation pathway, allowing pollen tube growth towards the ovaries where fertilization can take place^8^. Each *S*-allele encodes an S-RNase and multiple SLFs with different specificities, which together can recognize all S-RNases except the S-RNase that is present on the same allele^9^.

Although most diploid potato lines are self-incompatible (SI), self-compatible diploid potato lines do exist and can be used to introduce self-compatibility into diploid potato breeding programs^10–12^. Hosaka and Hanneman mapped a dominant *S*-locus inhibitor (*Sli*) gene from a *Solanum chacoense* accession at the distal end of chromosome 12 and used it to generate potato inbred lines^13,14^. Based on their results, Hosaka and Hanneman suggested that *Sli* is a pollen-expressed gene with sporophytic action and that homozygosity for *Sli* is lethal since homozygous *SliSli* genotypes were absent in the F8 population of *S. chacoense*. We used one of these *S. chacoense* (DS)-derived inbred lines to introduce self-compatibility into *S. tuberosum* backgrounds. Here, we describe the identification of the causal gene of self-compatibility to gain further insight into the biology of self-compatibility in diploid potato.

## Results and discussion

In one F2 population derived from a cross between the *Sli* donor (designated DS) and a diploid *S. tuberosum* (D2) we observed a modest effect QTL for self-berry set on chromosome 2, but a subsequent recombinant screening was not successful. We noticed that multiple F2 populations showed extreme skewness around the long arm of chromosome 12, with homozygosity for the non-DS haplotype being completely absent.

**Fig. 1:**
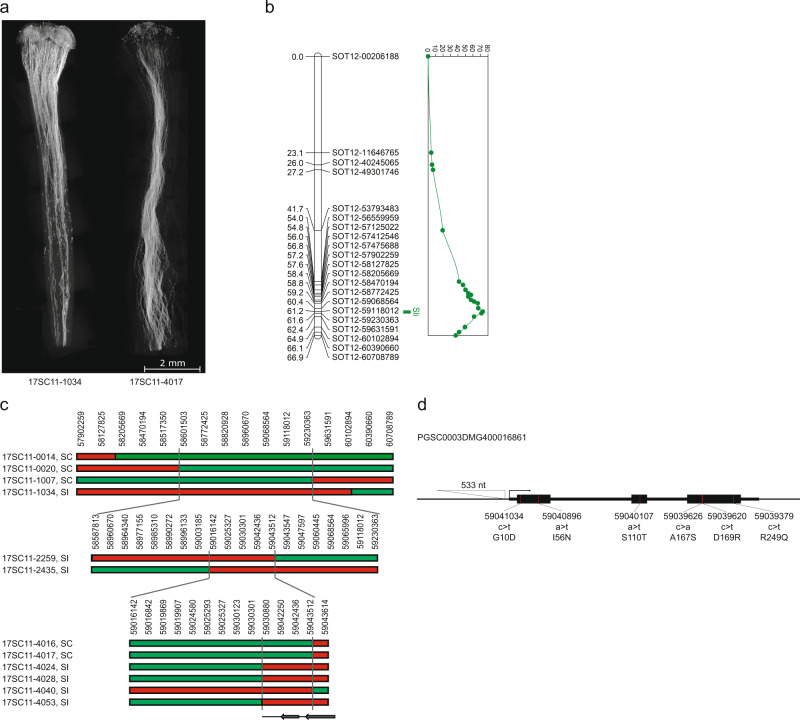
QTL mapping and recombinant analysis in population 17SC11. **a** Fluorescence microscopy of self-pollinated styles of SI (left) and SC (right) genotypes shows that self-pollen can grow through the styles of SC genotypes, while self-pollen tube growth is arrested in styles from SI plants. Although a few self-pollen tubes reach the end of the style in the SI genotype, these do not induce a berry set. These images are typical for SI and SC genotypes as evidenced in 50 or more similar genotypes. **b** The LOD profile resulting from QTL analysis in population 17SC11 shows a highly significant QTL on the distal end of chromosome 12. **c** Recombinant analysis suggests *Sli* must be located in an 12.6 kb interval, which contains two genes. The SC haplotype is shown in green, the SI haplotype is shown in red. Numbers above the haplotypes are the positions of the genotyped SNP markers on chromosome 12 of DM4.04. **d** Candidate gene PGSC0003DMG400016861 shows an SC-specific 533 nt insertion in the promoter, suggesting that the SC allele of PGSC0003DMG400016861 may show altered tissue-specific expression. The *Sli*-specific amino acid changes and their positions are shown below the gene model.

Based on this skewness and the mapping of *Sli* on chromosome 12, we hypothesized that *Sli* is gametophytically expressed, meaning that in a self-pollination of a plant heterozygous for *Sli* (*Sli*/*sli*), only pollen containing the dominant *Sli* allele can participate in self-fertilization. To test this hypothesis, we crossed a vigorous and highly self-fertile line (16HP1-66) to a vigorous self-incompatible line (D16), whole-genome sequenced both, and analyzed the resulting F1 population (17SC11, *n* = 251, Fig. 1a, b). As this F1 population is highly polymorphic for many loci, we observed a wide range of phenotypes, including those that are related to fertility. Therefore, we implemented a very strict and rigorous phenotyping protocol that includes berry and seed set from both cross and self-pollinations as well as visualization of pollen tube growth in styles to avoid issues of sterility confounding the compatibility phenotype. Plants that set more than one self-berry are considered SC, whereas plants that do not set self-berries after at least 10 self-pollinations, show self-pollen tube growth arrest in the style, and set cross-berries after pollination with bulked pollen are considered SI. As a result, a significant part of the population was excluded from genetic analyses as the requirements to unambiguously assess the compatibility phenotype were not met. Still, the compatibility status of the majority of the population 17SC11 progeny could be assessed and it was shown to segregate for self-compatibility (Supplementary Data 1). As self-compatibility originated from 16HP1-66, we used the whole genome sequences of this genotype to design KASP markers targeting SNPs on chromosomes 2 and 12 that are heterozygous in 16HP1-66 but homozygous in D16, enabling the mapping of *Sli* in the maternal meiosis. We constructed a genetic map, performed QTL analysis, and found a highly significant QTL (LOD = 75.72 ) on the long arm of chromosome 12 (Fig. 1b), confirming the results of Hosaka and Hanneman.

To confirm the QTL in a different genetic background, we crossed another highly self-fertile genotype derived from the Solynta breeding program with SI genotype D14 and analyzed the resulting F1 population (17SC25, Supplementary Data 1). Among 32 individuals of population 17SC25, we found no SI individuals. To generate a segregating population, we selected the most fertile genotype and crossed it to two SI genotypes that we identified in population 17SC11, resulting in populations 18SC11 and 18SC12 (Supplementary Data 1 and Supplementary Fig. 1). As expected, analysis of populations 18SC11 and 18SC12 showed that both populations segregate for self-compatibility. We submitted the mother (17SC25-8) for whole genome sequencing and used this data to design new KASP markers using the same approach as used for population 17SC11, but this time targeting only chromosome 12. Subsequent QTL analysis confirmed the QTL we found in population 17SC11 with LOD values of 33.14 and 120.94 in populations 18SC11 and 18SC12, respectively (Supplementary Fig. 2).

To determine whether *Sli* is indeed gametophytically expressed, we analyzed an F2 population (19SC1, *n* = 160) derived from a fertile and vigorous 17SC11 individual. Flowering and fertility in this population were reduced compared to the F1. In the phenotypic analysis, out of 160 plants, 81 plants were self-compatible, 78 were categorized as not determined (ND) due to poor flowering or poor fertility and one plant was scored self-incompatible (Supplementary Data 1). We designed KASP markers targeting SNPs on chromosome 12 that are homozygous for alternate alleles in parents 16HP1-66 and D16. Along the whole of chromosome 12 segregation ratios significantly deviate from the expected 1:2:1 segregation. Moreover, around the self-compatibility QTL, there are no loci homozygous for the haplotype of parent D16 (Supplementary Fig. 3), showing instead a 1:1 segregation for heterozygous D16/16HP1-66: homozygous 16HP1-66, suggesting that the elimination of pollen lacking *Sli* causes segregation distortion. This supports the hypothesis that only pollen carrying the dominant *Sli* allele participate in self-fertilization. In addition, apart from one individual, the phenotyping was conclusive for the contrast between SI and SC, showing that the used phenotyping protocol is robust and almost error-free.

While the 628 KB *SLI* interval on chromosome 12 carrying the *Sli*-allele from population 17SC11 was reduced to a smaller overlapping interval of 169 KB in population 18SC12, these intervals were still too large to identify the *Sli-*gene. Therefore, we aimed to reduce the *Sli*-containing interval via a recombinant screening approach. To identify plants with a recombination in the *Sli* interval, we genotyped 1374 17SC11 seedlings with two KASP markers on the proximal border and two on the distal border. We identified 81 seedlings with recombination between the two outermost markers and selected those for further fine mapping. To get unambiguous phenotypes, we vegetatively propagated these genotypes and performed the phenotyping on at least two clones per genotype. We genotyped the 81 recombinants with more markers in the interval and identified two informative recombinants that reduced the interval to 27.37 KB containing five annotated genes (Supplementary Data 1).

To further reduce the interval, we screened another 10165 seedlings from the same population with four markers around this 27.37 KB interval and identified 12 recombinants. These were further genotyped with 14 more markers in this interval and we identified six informative recombinants that showed clear compatibility phenotypes. Two recombinants with an SC phenotype and one with an SI phenotype confirmed the distal border of the 27.37 KB interval, while three recombinants with an SI phenotype defined a new proximal border that reduces the interval to only 12.6 kb containing two genes, PGSC0003DMG400016861 and PGSC0003DMG400016860 (Fig. 1c).

To identify the candidate gene that is responsible for the self-compatible phenotype, we analyzed the sequence variation for these two genes in several whole-genome sequenced diploid potato lines (Supplementary Data 2). By comparing this sequence variation with the SC/SI phenotypes of these lines, we identified all SC-specific SNPs and INDELS (Supplementary Data 2). Next, we manually identified all non-synonymous SNPs and determined whether the amino acid substitutions are common or unique for similar proteins in the Solanaceae. Candidate gene PGSC0003DMG400016861 shows six SC-specific amino acid substitutions and notably, a 533 bp insertion located at −108 bp from the start codon, suggesting that the SC allele has altered expression compared to the SI allele. Based on these genetic studies we hypothesized that PGSC0003DMG400016861 is the *Sli* gene.

**Fig. 2:**
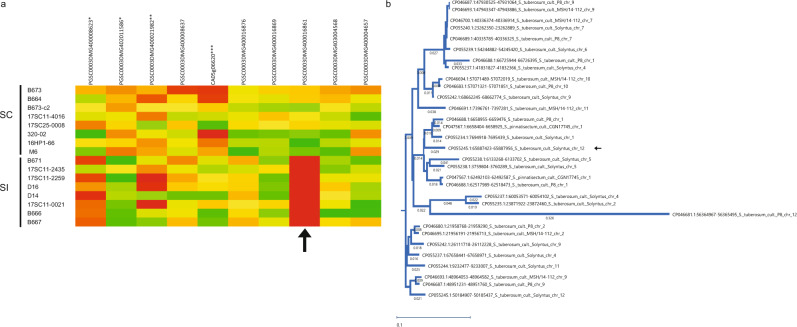
RNA sequencing of germinated pollen from SC and SI genotypes. **a** Expression of several pollen-expressed genes on chromosome 12. Candidate gene PGSC0003DMG400016861 (black arrow) is expressed in pollen from SC genotypes, but not in pollen from SI genotypes, whereas other pollen-expressed genes show similar expression levels in SC and SI genotypes. The color scale is based on FPKM values relative to the highest expressing genotype, ranging from green (highest expression) to red (lowest expression). * For these two genes the RNA-seq reads were mapped to the corresponding tomato gene model. ** For this gene the RNA-seq reads were mapped to the corresponding pepper gene model. *** Gene model CA05g06620 was inferred from Pepper and lacks a predicted counterpart in the PGSC gene catalog. **b** Phylogenetic tree of the 533 bp insertion and homologous sequences in potato. The 533 bp insertion in the SC allele of *Sli* is indicated with the arrow.

To verify the hypothesis that *Sli* is expressed in pollen, we germinated pollen from 10 SI and 10 SC potato genotypes in vitro and extracted RNA for RNA sequencing. From the two remaining candidate genes, only candidate gene PGSC0003DMG400016861 was expressed, but exclusively in pollen from SC genotypes (Fig. 2a). Furthermore, in plants heterozygous for the putative candidate *Sli* gene, only the *Sli* allele was expressed. Interestingly, other pollen-expressed genes located close to the *Sli* locus on Chromosome 12 showed similar expression levels in SC and SI plants (Fig. 2a). Therefore, we concluded that only the PGSC0003DMG400016861 gene is specifically expressed in pollen tubes of SC plants.

To investigate the origin of the 533 bp insertion, we performed a BLAST search of the 533 bp sequence on NCBI. Interestingly, sequences remarkably similar to the *Sli*-specific insertion are common in sequenced *S. tuberosum* accessions (Fig. 2b). Furthermore, the 533 bp insertion has an homology to a sequence in *S. pennellii*. Using the sequence from *S. pennellii* as a BLAST query we found similar sequences in *S. lycopersicum*. Phylogenetic analysis of the sequences in *S. tuberosum*, *S. pennellii* and *S. lycopersicum* groups the *S. pennellii* sequence together with the *S. lycopersicum* and one *S. tuberosum* sequence, suggesting that these share a common origin (Supplementary Fig. 4a). We hypothesized that the insertion is derived from a transposable element (TE). We generated a dot-plot graph from the 533 bp sequence and observed that the sequence contained miniature inverted repeats (Supplementary Fig. 4b). We submitted the 533 bp insertion to BLAST against the plant MITE database, resulting in multiple hits from MITE family DTA_Sot42 in *S. tuberosum*^15^, indicating that the 533 bp insertion in the promoter of *Sli* indeed originates from a TE (Supplementary Fig. 4c).

**Fig. 3:**
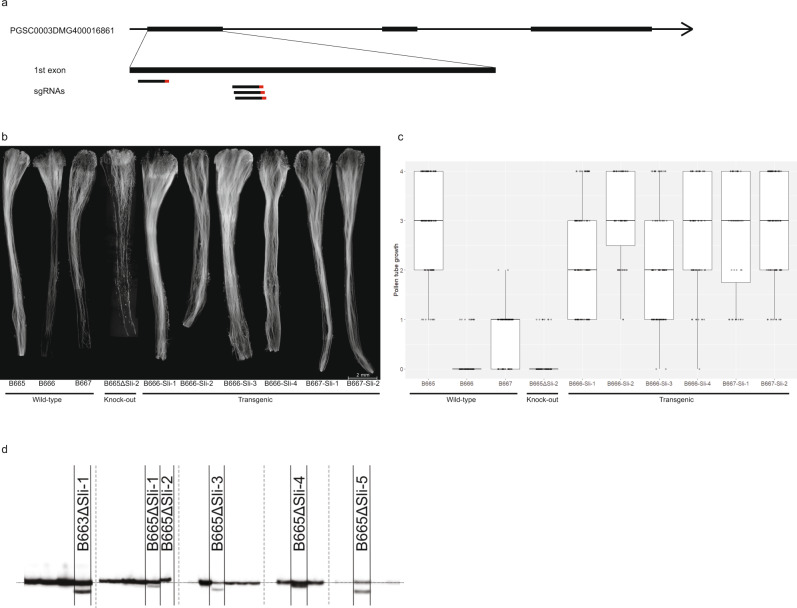
Phenotypic analysis of *Sli* transgenic and CRISPR–Cas9 knock-out lines. **a** Design of Sli pAGM:CRISPRΔSli construct. Four sgRNAs target the first exon of PGSC0003DMG400016861. Protospacer Adjacent Motifs are shown in red. **b** Fluorescence microscopy of self-pollinated styles from *Sli* transgenics, knock-outs, and untransformed controls. Wild-type B665 shows complete self-pollen tube growth through, but in knock-out line B665ΔSli-2 self-pollen tubes growth is arrested before the pollen tubes can reach the ovaries. Wild-type B666 and B667 show self-pollen tubes growth arrest, but transformation with pBINPLUS-Sli enables self-pollen tubes growth to the ovary. Each style in the image is composed of separate microscopy images of which the contrast and brightness levels were adjusted. The styles shown are representative of three independent experiments. **c** Pollen tube growth phenotypes of *Sli* transgenics, knock-outs and untransformed controls on a 0–4 scale: 0: no pollen tubes reach the ovary, 1: fewer than 25% of pollen tubes reach the ovary, 2: between 25 and 50% of pollen tubes reach the ovary, 3: between 50 and 75% of pollen tubes reach the ovary, and 4, more than 75% of pollen tubes reach the ovary. The boxes indicate the median and lower (25%) and upper (75%) quantiles, the whiskers indicate the smallest and largest observations. These data were generated in one greenhouse experiment with 3–5 clones of each genotype and are consistent in two independent experiments with the same genotypes. **d** PAGE analysis of PCR products from lines obtained after transformation of genotypes B663 and B665 with pAGM:CRISPRΔSli. Six regenerants show INDELs in the targeted area and are labeled B663ΔSli-1 and B665ΔSli-1 to B665ΔSli-5. The bands without labels are from regenerants in which pAGM:CRISPRΔSli did not induce INDELs. Additional lanes in the gel image were excised at the positions indicated by the vertical dashed lines. The horizontal dotted line represents a fragment size of 136 bp.

To further confirm that PGSC0003DMG400016861 is indeed *Sli*, we designed an expression construct containing the exons of the SC allele of *Sli* between its native promoter and terminator (Fig. 3a) in vector pBINPLUS (pBINPLUS-Sli). We used this construct to transform two SI genotypes from mapping population 18SC12. We phenotyped two to six clones from each of five independent transgenics derived from SI genotype B666, and three transgenics derived from the SI genotype B667.

Clones from six independent transgenics readily set berries upon self-pollination (Supplementary Data 3). In addition, fluorescence microscopy showed that pollen from *Sli* transgenic plants grows deeper into self-styles than untransformed controls (Fig. 3b, c). We scored pollen tube growth in 442 self-pollinations in *Sli* transgenics and 179 self-pollinations in untransformed controls on a 0–4 scale. The majority of pollen tubes reached the ovaries in most *Sli* transgenics, compared to only a very small fraction of the controls, indicating that PGSC0003DMG400016861 is the *Sli* gene.

Next, we designed a CRISPR–Cas9 construct encoding four gRNA’s targeting the first exon of PGSC0003DMG400016861 (pAGM:CRISPRΔSli, Fig. 3a). We transformed two SC genotypes (B665 & B663) with this construct and obtained 149 transformed regenerants. We then analyzed the targeted exon using PAGE to identify CRISPR–Cas9-induced INDELs. Unfortunately, the pAGM:CRISPRΔSli vector had a low efficiency, only six of the 149 regenerants showed INDELs in *Sli* (Fig. 3d). Five of these CRISPR–Cas9 lines are heterozygous for the INDELs, but one line, B665∆Sli-2, is homozygous for a small INDEL. While untransformed B665 readily sets self-berries and shows good self-pollen tube growth through 105 observed styles, B665∆Sli-2 does not set berries upon self-pollination and it’s pollen is not able to grow through 78 observed styles (Fig. 3b, c and Supplementary Data 3), providing further evidence that PGSC0003DMG400016861 is indeed the *Sli* gene.

In S-RNase-based gametophytic self-incompatibility systems, self-fertilization is prevented by pistil expressed S-RNases that enter pollen tubes and exert cytotoxic effects on self-pollen or any other pollen lacking a matching S-Locus F-box (SLF) protein. Cross-fertilization is enabled by pollen-expressed SLF proteins that can recognize and detoxify non-self S-RNases. Each S-allele encodes multiple SLFs that each of which can recognize a different S-RNase, and together can recognize most S-RNases present in potato except the S-RNase encoded on the same S-allele. *Sli* encodes an F-box protein PP2-B10, which consists of an F-box domain linked to a lectin domain. Lectin domains are known to interact with carbohydrates, and might be able to interact with glycosylated proteins^16^. Furthermore, S-RNases have been shown to be glycosylated^17^. We hypothesize that the 533 bp insertion in the promoter of the SC allele of *Sli* enables expression in pollen, where *Sli* is able to bind and detoxify self S-RNases, leading to loss of self-pollen tube growth arrest and thus self-compatibility. A detailed investigation into MITE activity in potatoes by Laimbeer found that 2% of MITE insertions close to genic regions were associated with alterations in gene expression. Furthermore, out of 1935 tested hAT insertions close to genic regions, 13 resulted in upregulation of the associated gene, indicating that the altered pollen-specific expression of *Sli* could indeed be caused by the 533 bp insertion in its promoter^18^. Nevertheless, further research is required to determine the validity of this hypothesis.

Previously, Clot et al used a bulked segregant K-mer mapping approach to identify a 333 kb interval on chromosome 12 in which *Sli* must be located^19^. Here, we mapped the *Sli* locus to the same region of chromosome 12 in an F1 population and used a recombinant screening to reduce the interval to 12.6 KB containing 2 genes. Expression analysis revealed that the SC allele of one of these genes is specifically expressed in pollen from SC genotypes. Finally, using transgenic expression and CRISPR–Cas9-induced knock-out, we conclusively show that PGSC0003DMG400016861 is *Sli*. While the study by Laimbeer showed that MITEs can upregulate proximate genes in a tissue-specific manner, more research is required to prove that the presence of the MITE in the *Sli* promoter is the cause of its pollen-specific expression.

In the materials investigated in this study (Supplementary Data 4), we could not confirm earlier reports of lethality associated with homozygosity for *Sli*, since we found viable F2 plants homozygous for *Sli* that were able to set berries (Supplementary Data 1)^12,20^. In addition, the genome sequences of the inbred-line Solyntus, as well as the inbred-line M6, show that both these lines are homozygous for *Sli*, indicating that homozygosity for *Sli* itself is not lethal, although it is still possible that a lethal allele genetically linked to *Sli* in an ancestor has been removed via recombination in these genotypes^21,22^. However, from the data generated in this study, we cannot exclude the possibility that the segregation distortion observed in the F2 population is caused by a lethal allele linked in phase to the SI allele of *Sli*. As yet, it is unclear whether *Sli* itself can directly recognize and detoxify S-RNases. Further, it is not clear whether *Sli* results in self-compatibility in all S-locus genotypes. It is possible that the function of some S-alleles cannot be inhibited by *Sli*. Further research is needed to resolve this question.

While the identification of *Sli* further enables inbred-line-based hybrid breeding using diploid potato, other hurdles remain. First, most significantly, diploid potato suffers from inbreeding depression, which leads to reduced vigor and fertility upon inbreeding. Purging of deleterious alleles by continued self-fertilization of diploid potato lines is an efficient method to reduce inbreeding depression and has already resulted in the generation of comparatively vigorous and fertile potato inbred lines^2,23–25^. Second, the donor of *Sli* used in this study, DS, is derived from a *S. chacoense* accession, potentially leading to issues with linkage drag of deleterious alleles from *S. chacoense*. In the diploid breeding program of Solynta, we do not see apparent issues due to deleterious alleles coming from *S. chacoense*. Moreover, a recent study on self-compatibility revealed that SC-specific k-mers are already present in several tetraploid cultivars, providing a route to circumvent this potential linkage drag altogether by using dihaploids generated from these cultivars as *Sli* donors^19^.

## Methods

### Plant materials

All used plant materials are listed in Supplementary Data 4

### Greenhouse conditions

All plants were grown in greenhouses that were heated when the temperature dropped below 14 °C and cooled by opening the windows when the temperature increased above 19 °C. Artificial lighting supplemented the natural light when the light intensity dropped below 85 W/M2. Plants were grown in a special potato substrate mix from Lentse Potgrond (Lentse Potgrond B.V, Katwijk, the Netherlands). The substrate mix used is composed out of a peat-mixture for balanced water uptake, basic slow-release fertilizer, and lime to ensure the required pH level. The substrate mix was fertilized using a 20:20:20 Nitrogen:Phosphorus:Potassium solution with an electrical conductivity (EC) of 1.5.

### Evaluation of self-compatibility

Flowers and buds were counted once a week and vigor was scored once per month on a scale from 1 to 9 with 1 being an extremely non-vigorous plant, and 9 being an extremely vigorous plant. Pollen from multiple flowers from one plant was collected in an Eppendorf tube and used immediately for self-pollination on the same flowers with a maximum of 10 flowers per plant per week. Plants that set more than two self-berries containing at least 35 seed per self-berry were classified as self-compatible. To determine female fertility, plants were pollinated with bulked pollen from at least three unrelated genotypes. Plants that did not set self-berries after at least 10 self-pollinations, but did set at least one bulk berry and showed fertile pollen in microscopic analysis of self-pollinated styles were classified as self-incompatible. For 40 genotypes from the mapping populations in which the berry and seed set data were inconclusive (17SC11: *n* = 14, 18SC11: *n* = 7, 18SC12: *n* = 19), phenotypical classification was based on self-pollen tube growth through the styles (noted in Supplementary Data 1).

### Style imaging

To visualize pollen tube growth, pollinated styles were removed 24–48 h after pollination and then fixed in 3:1 ethanol:acetic acid for at least 24 h. The styles were then macerated in 8 M NaOH for 10 min at 65 °C and rinsed twice with deionized water. Styles were placed on microscopy slides and stained for 2–5 min using 0.1% Aniline blue (Carl Roth GmbH) in 0.1 M K_4_P_2_O_7_ (pH = 7), then squashed in glycerol using a coverslip and observed using a Zeiss Axiolab fluorescence microscope using a filter set 01 (BP 365/12, FT 395 and LP 397). All styles were observed and scored using two parameters: (1) deepest penetration into the style, as expressed in percentage of maximal penetration, (2) % of pollen tubes reaching the deepest penetration. We then converted these percentages to a 0–4 scale, where styles in which no pollen tubes reached the ovary got a score of 0, styles in which between 0 and 25% of pollen tubes got a score of 1, styles in which between 25 and 50% of pollen tubes reached the ovary got a score of 2, styles in which between 50 and 75% of pollen tubes reached the ovary got a score of 3, and styles in which more than 75% of pollen tubes reached the ovary got a score of 4.

### Image acquisition

Selected styles were imaged using a Zeiss Axiophot microscope with filter set 01, using a Zeiss AxioCam ICc 5. The images were made using the Zeiss Zen 2.3 (blue edition) software package. During acquisition, the settings were adjusted to minimize background. Styles were imaged using the ×5 objective and were saved as TIFF files with a resolution of 2464 × 2056 pixels with 24-bit depth. Up to eight separate images were then assembled using Panavue image assembler. The contrast and brightness of the assembled styles were adjusted to create Figs. 1a and 3c.

### DNA extraction

For KASP analysis of the mapping populations, leaf samples were sent to VHLGenetics (Wageningen, The Netherlands) for DNA extraction using sbeadex™ kits (LGC Genomics GmbH, Berlin, Germany) according to the protocol supplied by the manufacturer.

### KASP analysis

Kompetitive allele-specific PCR (KASP™) analysis was performed by VHLGenetics (Wageningen, The Netherlands) using KASP assays designed to be specific for SNPs that segregate in our material. KASP assays were conducted according to the protocol supplied by the manufacturer (LGC Genomics GmbH, Berlin, Germany). The results from the KASP assays were visualized using SNPviewer (available at lgcgroup.com/products/genotyping-software/snpviewer) to confirm correct segregation and genotype calling.

### Linkage analysis

Haplotypes of self-compatible female parents were reconstructed from the genotype data by analyzing recombination rates between different SNPs. This data was used to convert the SNP calls into an “axb” format, wherein the “a” haplotype is linked to the self-compatible allele of *Sli*, while the “b” haplotype is linked to a self-incompatible allele of *Sli*. Linkage maps were created using Joinmap 4.1^26^ with population type DH and default settings.

### QTL mapping

The phenotype data was converted to a numerical trait by assigning 1 to each self-compatible genotype, 0 to each self-incompatible genotype, and * to genotypes for which compatibility could not be determined. QTL mapping was performed using interval mapping in MapQTL^27^. The outputs of MapQTL were used to generate QTL plots with Mapchart 2.3^28^.

### Bioinformatic analysis

To identify correct gene models in the initial 27.37 kb interval, we investigated two separate gene annotations for the DM4.04 reference genome, the PGSC annotation, and the ITAG annotation. See also Hirsch et al.^29^. To confirm the correctness of the annotations, we performed BLASTp searches with the predicted protein sequences from both annotations. By comparing the best hits in the BLASTp search to our query, we determined whether all annotated exons and domains in the predicted protein sequence were supported by similar proteins in potato and other plant species. Furthermore, publicly available RNA-seq libraries on SPUD DB (available at solanaceae.plantbiology.msu.edu/cgi-bin/gbrowse/potato/) and NCBI genome data viewer (available at ncbi.nlm.nih.gov/genome/gdv/browser/) were used to determine whether putative exons had expression evidence. Together, these two approaches allowed us to validate the intron–exon structures of the gene models in both annotations, resulting in an informed choice for one or more isoforms of gene models to represent the gene in question. Based on these approaches, candidate gene PGSC0003DMG400016862 was recognized as likely partial and insignificantly expressed and discarded from further analyses. The gene model Sotub12g029970 was deemed correct, while its PGSC counterpart PGSC0003DMG400016860 is likely truncated. Because it is located largely outside the designated interval, and no relevant amino acid substitutions between SC and SI plants could be identified, this gene was discarded from further analyses.

### Variation analysis

To identify mutations in the 27.37 kb interval that are specific for self-compatible genotypes all high confidence SNPs (Supplementary Data 3) were determined that were (1) homozygous in DS, 17SC100-18 and 17SC100-2 (because all three are homozygous for the SC allele of *Sli* (*Sli/Sli*)), (2) homozygous different in D16 (because D16 is homozygous for the SI allele of *Sli* (*sli/sli*)), and (3) heterozygous in both 16HP1-66 and 17SC25-8 (because both are heterozygous for SC allele *Sli* (*Sli/sli*)). The allelic sequence was obtained by de novo assembly using SPAdes version 3.11.1^30^ of 150 nt paired-end Illumina data of the above-listed plants (of approximately 25–30× sequencing depth). Resulting contigs were aligned to the DM reference (using minimap2 version 2.1) and filtered for those reliably aligning to the 27 kb. From these aligned contigs, variation relative to DM4.03 was quantified straightforward (using the subroutines mpileup and call from bcftools, version 1.9) and listed in the Variant Call Format (VCF).

### Amino acid change analysis

From this list of SC-specific mutations, all non-synonymous SNPs were identified by overlapping with the designated coding exons. The amino acid changes relative to either DM or SI sequence were listed. Unique amino acid changes were identified by performing BLASTp searches using the protein sequence and performing multiple sequence alignment using the top 100 BLASTp hits.

### Variation in promoter and terminator regions

The promoter region was chosen to be the sequence upstream of the start codon until the coding sequence of the upstream gene with a maximum of 1500 nt. Dramatic variation in promoter regions was found within the 27.37 kb interval, of which most striking were several larger deletions and insertions of tens to hundreds of nucleotides of length. All variation in the *Sli* interval, relative to DM, was obtained, including that of the promotor/upstream region as well as the terminator/downstream region.

### Pollen acquisition and germination

Pollen from the genotypes listed in Fig. 2a was obtained by vibrating open flowers using an electronic toothbrush and collecting the pollen in 1.5 ml Eppendorf tubes. After the acquisition, the pollen was dried by storing the open Eppendorf tubes with pollen in an air-sealed box containing silica gel for 24 h at room temperature. Afterward, the pollen was stored at −20 °C until further use.

Pollen was germinated by suspending 2.5 mg of dried pollen in 5 ml of liquid medium (9% (w/v) sucrose, 50 mg/l Boric acid, 73.5 mg/l CaCl_2_·2H_2_O, 118 mg/l Ca(NO_3_)2·4H_2_O, 123 mg/l MgSO_4_·7H_2_O) in 3.5 cm diameter Petri dishes sealed with parafilm. The pollen was left to germinate in the Petri dishes for 24 h in the dark in a shaking incubator at room temperature and shaking at 125 RPM. The liquid medium containing the germinated pollen was then carefully pipetted into 2 ml Eppendorf tubes using pipette tips that were modified to increase the aperture size so as not to damage the pollen tubes. The Eppendorf tubes were then centrifuged at 600×*g* for 1 min and the medium was carefully removed by pipetting. The pellet and some remaining medium were then immediately frozen in liquid nitrogen, two stainless steel beads (2 mm diameter) were added and the samples were grinded using a TissueLyser II (Qiagen GmbH, Hilden, Germany) at 20 Hz for 1 min.

### RNA extraction and sequencing

Buffer RLT (Qiagen GmbH) was added to the grinded pollen samples while making sure that the samples remained frozen. RNA extraction was then performed using the RNeasy mini kit according to the manufacturer’s protocol (Qiagen GmbH, Hilden, Germany). The 250–300 bp insert-size cDNA libraries were sequenced as 150nt paired-end reads, yielding 30–42 million read-pairs per sample (Novogene, Cambridge, United Kingdom).

### Other RNA-seq data sets

To create an overview of (tissue-specific) expression levels, all paired-end sequenced RNA-seq data sets tagged as ORGANISM “*Solanum tuberosum*” were downloaded from the public domain (NCBI-SRA, date 2018/17/13), totaling 441 paired fastq data sets. From these 441 public data sets, 3 were generated from style tissue (SRR7402817-SRR7402819) and all others from various non-pollen tissues, developmental stages, and accessions of plants.

### Solyntus reference assembly

For expression analyses, the recently acquired draft assembly of homozygous reference line Solyntus (version 1.0, downloadable at www.plantbreeding.wur.nl/Solyntus/) was used as a reference genome. Solyntus is an essentially homozygous variety generated as part of the breeding program of Solynta^21^. The mapping intervals in this study were inferred from the DM v4.03 genome assembly^31^ to the Solyntus 1.0 genome assembly by basic similarity searches (using BLASTn and bedtools) to be located at (Solyntus 1.0 genome assembly coordinates) 53532708–53954293 (Interval I, 421.6 kb < −628.9 kb), 53683239–53867377 (Interval II, 184.1 kb < −168.7 kb), 53731620–53763003 (Interval III, 31.4 kb < −27.4 kb) and 53753977–53763003 (Interval IV, 9.0 kb < −12.6 kb), respectively. In between brackets are the consecutive mapping interval number [Solyntus 1.0 coordinates], size in Solyntus-1.0, and size in DM-4.03/4.04, respectively. All intervals are located on chromosome ST4.03ch12_RaGOO (being chromosome 12) and do not contain a single gap in the Solyntus 1.0 assembly. Interval size variation is caused by a multitude of gaps (N’s) in the corresponding DM sequence and extensive variation between both genomes. Corresponding intervals on DM genome (DM-4.03/4.04): Interval I: chr12:58601503–59230363, Interval II: chr12:58962004–59130723; Interval III: 59016142–59043512; Interval IV: chr12:59030880–59043512.

Gene annotation on Solyntus 1.0 was inferred from three distinct gene catalogs (potato DM4.03, ITAG4.0 Tomato Genome Annotation Release of Sep 6, 2019^32^, and Pepper-v. 1.55^33^), which were mapped onto the Solytus assembly by using GeMoMa (v1.6.1). This was done to compensate for imperfections in individual gene catalogs and maximize our awareness of the existence of possible genes and/or expressed loci.

### RNA-seq read-mapping and transcript abundance quantification

All 5 SC, 3 SI, and all 441 public RNA-seq data sets were mapped to the Solyntus reference genome using hisat2 (version 2.1.0). The hybrid gene catalog obtained using GeMoMa was used for transcript-guided abundance estimation using StringTie (version 2.1.1) with settings -t -c 5 -f 0.05 -G and a GeMoMa concatenated Solyntus1.0 gff file. All observed expression in a 500 kb interval surrounding the *Sli* locus as a center was evaluated, in which interval a total of 90 (inferred) gene loci are located. We confirmed the absence of any noticeable expression in SC samples outside of any of these gene loci. In the 500 kb interval, we indicated the subsequently smaller number of candidates genes when intersecting with our mapping intervals I–IV as defined above.

### Confirmation of haplotype-specific expression

From 90 expressed loci in the 500 kb interval, only 8 were expressed above a selected threshold of 20 FPKM in all of the SC/SI samples. We used these sites to measure haplotype-specific (*Sli* or *sli*) expression level differences. The expression threshold selected enabled sufficient read depth to eventually and reliably phase the expression into (at most) 2 haplotypes. Together with the PSC locus itself (which lacks expression in SI plants), these 8 + 1 loci were haplotyped in each of the 8 samples (SAMtools phase version 1.7, default settings). The resulting haplotyped (paired) fastq files were de novo assembled using SPAdes (version 3.11.1). The resulting contigs were filtered for ample abundance and presumed full-length mRNAs, corresponding to the main (haplotyped) expressed isoform. In some cases, this removed alternatively spliced isoforms, none of which were supported by ample reads to be of any obvious biological importance. The variation in these haplotyped mRNA sequences was used to (dis)confirm if one or both haplotypes were expressed in each of the corresponding loci/samples.

### Design of *Sli* expression construct

We used the sequence of the *Sli* donor plant DS to design the *Sli* expression cassette. To allow native expression of *PSC*, we constructed a nucleic acid sequence comprising the native promoter (1563 bp upstream of the start codon), the three exons, and the native terminator (740 bp downstream of stop codon). Thus, both introns were removed from the *PSC* gene of donor plant DS. This sequence was synthesized and cloned into pBINPLUS by Genscript (Genscript Biotech, Leiden, the Netherlands). We refer to the vector containing the *Sli* insert as pBINPLUS-Sli.

### Construction of CRISPR–Cas9 vector

We designed four gRNAs based on the sequence of PGSC0003DMG400016861 in DM4.03 in locations in which no variation between the SC and SI alleles was present. For the selection of suitable guides and construction of the vector, we used the method described by Santillán Martínez et al.^34^. Briefly, four sgRNAs were selected according to the guidelines described by Liang et al.^35^. The CC-Top CRISPR/Cas9 target prediction tool was used to generate a list of sgRNAs^36^, folding was assessed using the Mfold web server^37^, and activity of the sgRNAs was predicted using the sgRNA scorer^38^. The following guides were selected and used to construct the vector pAGM:CRISPRΔSli: exon5.1T01 (ATTTCATCCGCGATCTCTCGGGG), exon5.1T04 (GATTTCATCCGCGATCTCTCGGG), exon5.1T06 (TATTTCCTATTGCTACCAGAAGG), and exon5.1T07 (TGATTTCATCCGCGATCTCTCGG). The CRISPR construct was then synthesized using plasmids obtained from Addgene: pICH86966 (template for amplification); pICSL01009 (level 0 plasmid); pICH47751, pICH47761, pICH47772, pICH47781 and pICH47732 (level 1 plasmids); pICH41822 (linker for four guides); and pAGM4723 (level 2 binary vector). The plasmid was cloned using *E. coli* DH5α and purified plasmid was sent for sequencing using primers PDS5843 (TTTGTGATGCTCGTCAGGGG), PDS8535 (CCCGAGAATTATGCAGCATTTT) PDS8536 (TCATCAGTCAATTACGGGGCT), and AL717 (GCTTGGCATCAGACAAACCGG) to confirm the presence and correct orientation of all components (NPTII, Cas9, and sgRNAs).

### Transformation of pBINPLUS-Sli and the pAGM:CRISPRΔSli vector into *Agrobacterium tumefaciens*

We transformed pBINPLUS-Sli into *A. tumefaciens* strain AGL0 and pAGM:CRISPRΔSli into *A. tumefaciens* strains AGL0 and AGL1 using an electroporation protocol. We took 40 µl of competent AGL0 cells and added 110 µl of ice-cold milliQ water. We pipetted 50 µl of this mixture into pre-cooled Eppendorf tubes on ice and added 1 µl of the plasmid. We left the cells on ice for 15 min and transferred the cells to pre-cooled electroporation cuvettes. We electroporated the mixtures with a Micropulser™ (Bio-Rad Laboratories, Veenendaal, the Netherlands) using the program Ec1 (1.8 kV, 0.1 cm cuvette). We added 1 ml of LB and incubated the cells for 3 h on a shaker at 28 °C and 200 RPM. Afterward, we inoculated LB agar plates containing Rifampicin (100 µg/ml) and Kanamycin (50 µg/ml) with the transformation culture. All picked colonies were confirmed to contain the correct vector.

### Transformation of potato genotypes

We transformed genotypes B666 and B667 with pBINPLUS-Sli and genotypes B663 and B665 with pAGM:CRISPRΔSli vector using the stem explant method described by Visser^39^. Briefly, internode explants were obtained from in vitro grown genotypes and were put on Petri dishes containing R3B medium with 2 ml of PACM medium. The next day, 50 ml of 48 h *Agrobacterium* cultures were centrifuged and resuspended in 75 ml of LB. The internode explants were then submerged in the *Agrobacterium* suspension for 5 min, dried on the filter, and placed back on the Petri dishes containing R3B medium. After 48 h of incubation, the explants were transferred to Petri dishes containing MS20 with antibiotics and placed in a growth chamber to allow regeneration of shoots. After regeneration, the shoots were grown on MS20 media containing cefotaxime (200 µg/ml), vancomycin (200 µg/ml), and kanamycin (100 µg/ml). When the shoots reached sufficient length, cuttings were made and grown in MS20 without antibiotics. After at least two weeks of growing on MS20 without antibiotics, the plants were planted in the greenhouse.

### Ploidy analysis

The ploidy of transgenic plants as well as the non-transformed controls was determined using flow cytometry by Plant Cytometry Services (Didam, the Netherlands). All tetraploid regenerants were discarded.

### PAGE analysis of CRISPR–Cas9-induced mutations

DNA extraction, PCR, and PAGE analysis were performed by Limgroup (Horst, The Netherlands). The following primers were used to amplify the CRISPR–Cas9-targeted region: Forward primer: CTATTTCCTATTGCTACCAG, reverse primer: AAACTTTACCCAAATAACGTC. Labeling of the PCR products was achieved by adding a reverse primer with an M13 tail (primer sequence: TGTAAAACGACGGCCAGTAAACTTTACCCAAATAACGTC) and either 700 IRDye or 800 IRDye labeled M13 primer into the PCR mixture. The resulting PCR products were analyzed on PAGE using a Li-cor system. PCR products from most of the lines without CRISPR–Cas9-induced mutations were excised from the gel images to produce Fig. 3d (excisions indicated by dashed lines).

### Phylogenetic analysis of the 533 bp insertion

The sequence of the 533 bp insertion was analyzed using BLASTn on the NCBI website. The best 30 hits, including the insertion present in *Sli* in Solyntus, were then downloaded and aligned in MegAlign Pro 17 (DNASTAR) using MUSCLE with default settings. The trees were generated using the neighbor-joining algorithm with default settings.

### Reporting summary

Further information on research design is available in the Nature Research Reporting Summary linked to this article.

## Supplementary information

Supplementary Information

Descriptions of Additional Supplementary Files

Supplementary Data 1

Supplementary Data 2

Supplementary Data 3

Supplementary Data 4

Reporting Summary

## Data Availability

The Solyntus genome sequence and raw sequence reads are available on NCBI under accession PRJNA631911. The Solyntus genome assembly and annotation files are available from WUR [https://www.plantbreeding.wur.nl/Solyntus/]. The RNA sequencing data from germinated pollen is available on the NCBI short read archive under accession PRJNA713577. Other data are available in the source data file or will be made available upon request. Source data are provided with this paper.

## Source data

Source data
