# Supplementary material for: Neofunctionalisation of the Sli gene leads to self-compatibility and facilitates precision breeding in potato
Source: Nat Commun. 2021 Jul 6;12:4141. doi: 10.1038/s41467-021-24267-6 (PMC8260583; doi:10.1038/s41467-021-24267-6)
Supplement: Supplementary file 1 — Supplementary Information [file 41467_2021_24267_MOESM1_ESM.pdf]

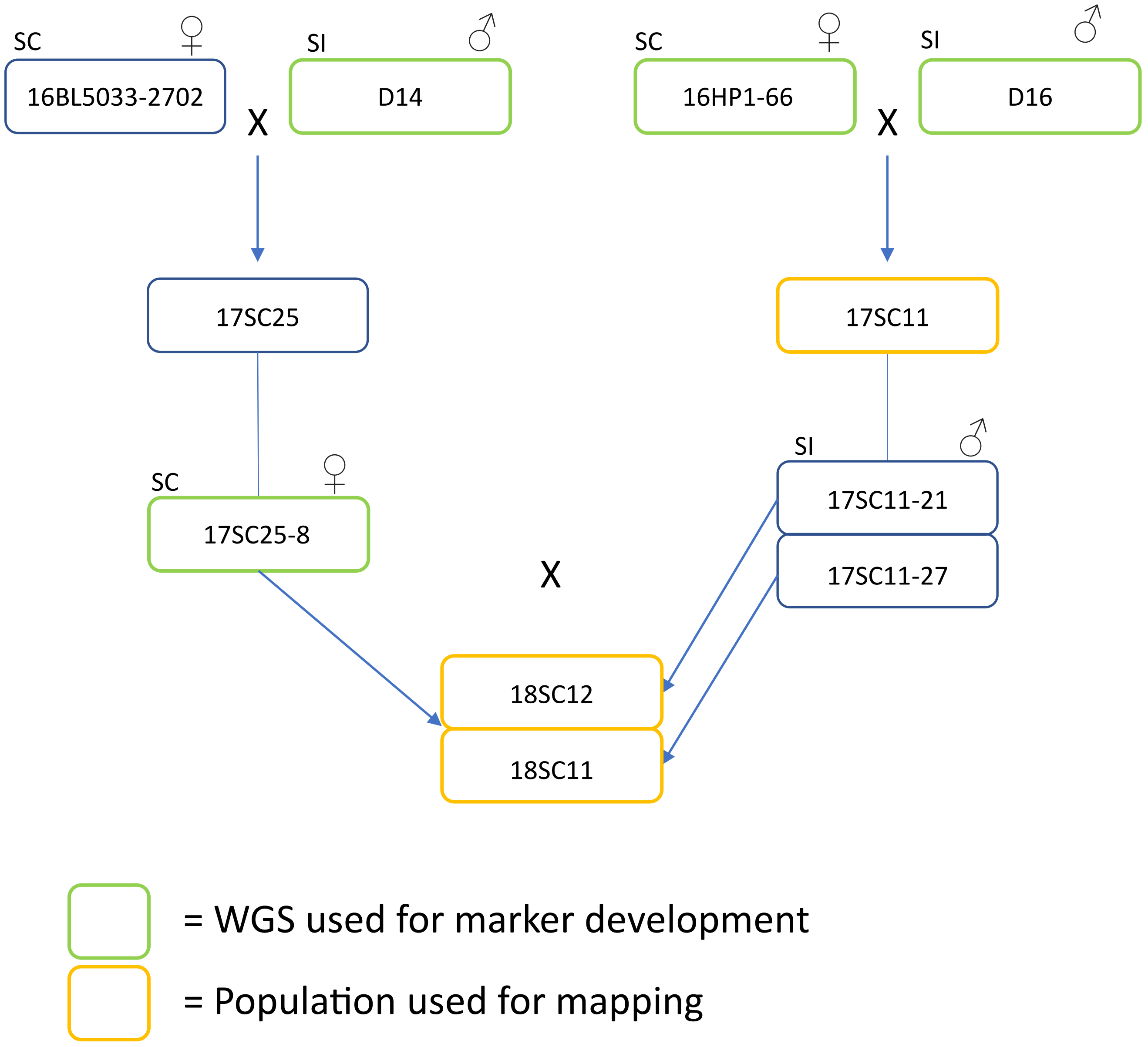

Supplementary Figure 1. Pedigree of mapping populations. Genotypes highlighted in green were sequenced to enable marker development. Populations highlighted in yellow were used for QTL analysis.

a

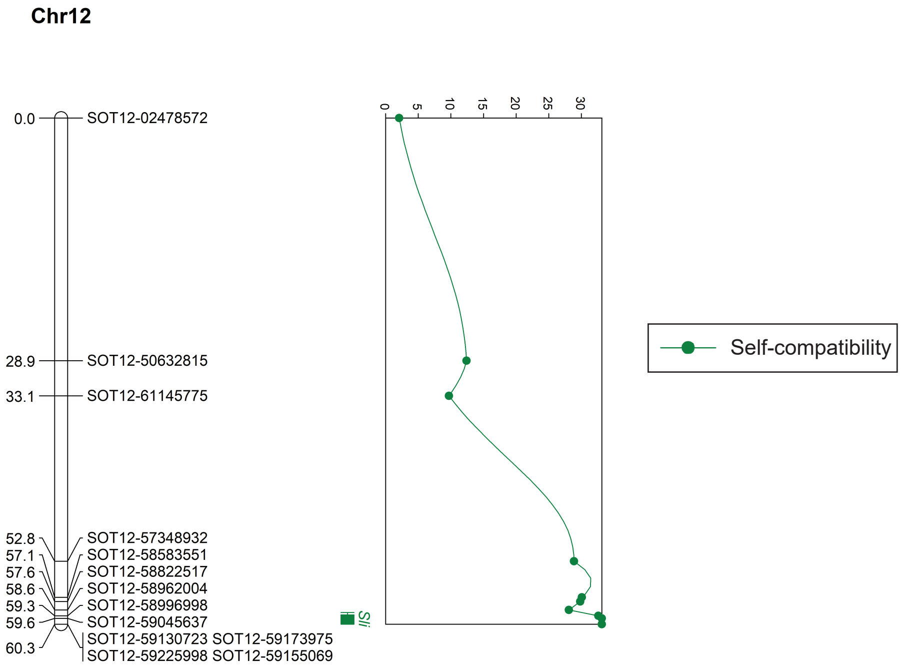

b

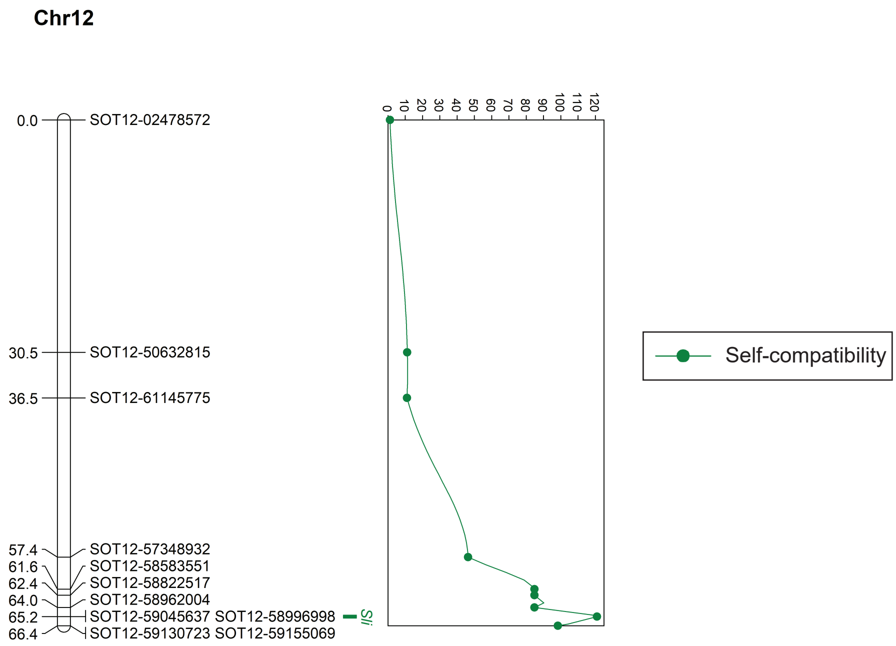

Supplementary Figure 2. QTL analysis in populations 18SC11 and 18SC12. a. LOD profile of QTL analysis for self-compatibility in population 18SC11. Green line shows LOD score. The 1-LOD and 2-LOD intervals are highlighted between the genetic map and the LOD profile. **b.** LOD profile of QTL analysis for self-compatibility in population 18SC12. Green line shows LOD score. The 1-LOD and 2-LOD intervals are highlighted between the genetic map and the LOD profile.

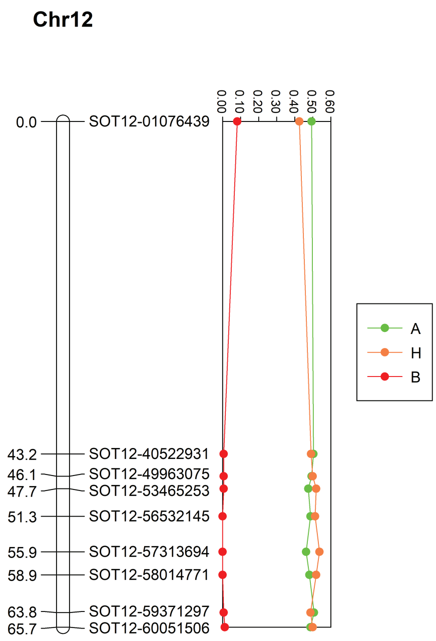

Supplementary Figure 3. Frequency of genotypes observed in the F2 population 19SC1 (n=160). The proportion of plants homozygous for the HP66 haplotype are shown in green. The proportion of plants heterozygous for the HP66 and D16 haplotypes are shown in orange. The proportion of plants homozygous for the D16 haplotype are shown in red.

a

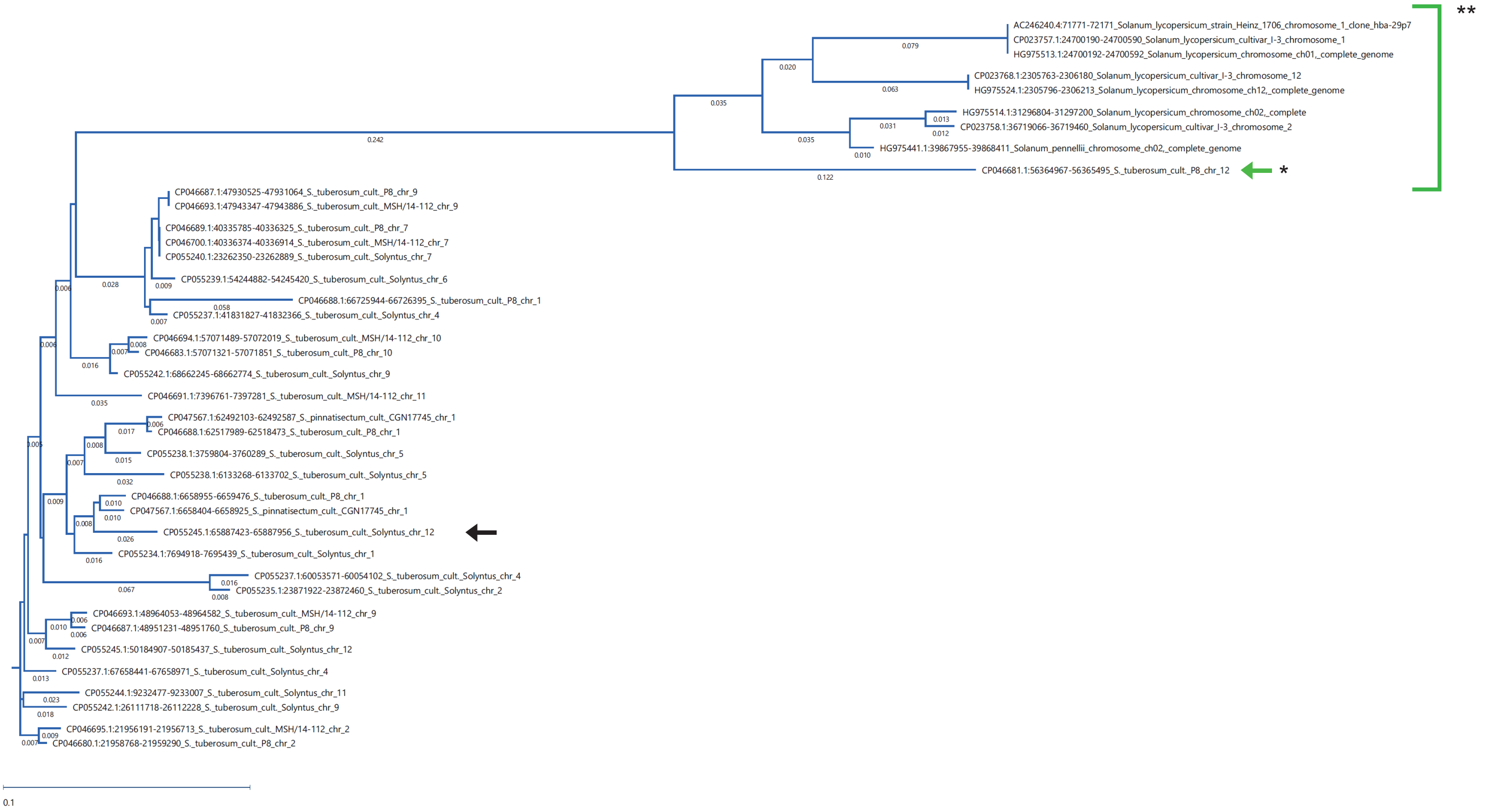

b

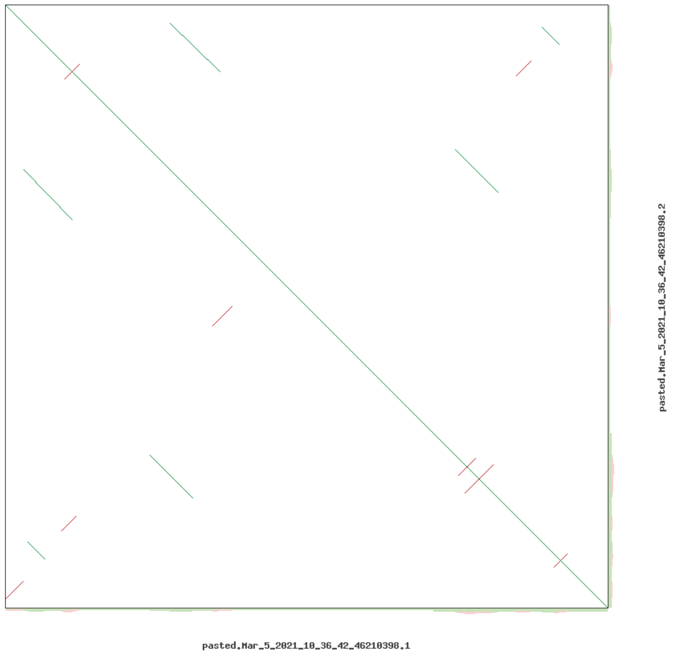

c

```
>SQ383018181 Solanum tuberosum, chromosome:chr01, from MITE family DTA_Sot42,
          SuperFamily hAT, partial sequence
         Length = 525

 Score =  795 bits (401), Expect = 0.0
 Identities = 497/525 (94%), Gaps = 3/525 (0%)
 Strand = Plus / Plus


Query: 1   caggggcggctcaacgtatttggaggcctaaaacaaaatttaaattaaaggcctaaaatc 60
           |||||||||||||||||| |||||||||| || |||||||||||||||||||||||||||
Sbjct: 1   caggggcggctcaacgtacttggaggcctgaagcaaaatttaaattaaaggcctaaaatc 60


Query: 61  ttttagctgaggcaattattaaataaattgttaacattattctataagtaataagttgac 120
           ||||||||||||||||||||| ||||||||||||||||||||||||||||||||||||||
Sbjct: 61  ttttagctgaggcaattattatataaattgttaacattattctataagtaataagttgac 120


Query: 121 aaaactgcttataaacttctttttttatttaaaagcacataacataagtcaatctaaaca 180
           ||||||||||||||||||||||||| |||||||| ||||||| |||| |||||||||| |
Sbjct: 121 aaaactgcttataaacttctttttt-atttaaaaacacataaaataaatcaatctaaata 179


Query: 181 ggcttgtaattcgctttatccaacacattagttttactattgattcatatttttgataga 240
            |||||||||| |||||||||||||||| | |||||||||||||||||||||| ||||||
Sbjct: 180 agcttgtaatttgctttatccaacacatgatttttactattgattcatattt-gataga 238


Query: 241 gctctaacttacatagagtataaaaggggtatagaaaattacaacgcgagagtaagtgaa 300
           |||||||||||||||||||||||||| ||| |||||||||||||||||||||| ||||||
Sbjct: 239 gctctaacttacatagagtataaaagtggtctagaaaattacaacgcgagagttagtgaa 298


Query: 301 gagagtgtaagaagacaaaacaacgtttttcttgatttcttctatttgattgaggttaag 360
           |||| ||||| ||||||||||||| |||||||||||||||||||||||||| ||||||||
Sbjct: 299 gagattgtaaaaagacaaaacaacatttttcttgatttcttctatttgatttaggttaag 358


Query: 361 gagaataaaataatatatatatgaaaagtacatttatcttaaataattaattttttctat 420
           |||||||||||  |||||||||||||| ||||||||||||||||||||||||||||||||
Sbjct: 359 gagaataaaattttatatatatgaaaaatacatttatcttaaataattaattttttctat 418


Query: 421 aaaaaaaattaacacataatttattgttggtaaaaatttgaggcccccctaaaattgggg 480
           ||||||||||||||||||||||||| ||||||||||||||||| ||||||||||||||||
Sbjct: 419 aaaaaaaattaacacataatttattattggtaaaaatttgagg-ccccctaaaattgggg 477


Query: 481 gcctaaggcatatgcctaatttttataagcattgagccggcactg 525
           ||||||||||||||||||||||||||||||||| |||||| ||||
Sbjct: 478 gcctaaggcatatgcctaatttttataagcattcagccggtactg 522
```

Supplementary Figure 4. Analysis of the 533 bp insertion in the *Sli* promoter. **a**. Phylogenetic tree of sequences similar to the 533 bp insertion including similar sequences in *Solanum lycopersicum*. Interestingly, one *S. tuberosum* sequence (*) is grouped together with the *S. pennellii* and *S. lycopersicum* sequences (**). **b**. Dot-plot analysis of the 533 bp insertion showing the presence of miniature inverted repeats. **c.** Alignment of the 533 bp insertion with the highest score BLAST hit on the pMITE database showing high identity between the insertion and a MITE family DTA_Sot42 TE from S. tuberosum.
