# Supplementary material for: Neofunctionalisation of the Sli gene leads to self-compatibility and facilitates precision breeding in potato
Source: Nat Commun. 2021 Jul 6;12:4141. doi: 10.1038/s41467-021-24267-6 (PMC8260583; doi:10.1038/s41467-021-24267-6)
Supplement: Supplementary file 2 — Descriptions of Additional Supplementary Files [file 41467_2021_24267_MOESM2_ESM.pdf]

Descriptions of Additional Supplementary Files

Supplementary Data 1
Description: Genotype and phenotype data of the mapping populations (17SC11, 18SC11, 18SC12 & 19SC1). For each population, the first sheet lists the genotypes and fertility data. The following lists shows the microscopic analysis of pollen-tube growth.

Supplementary Data 2
Description: Sequence variants of the Sli SC-specific haplotype, compared to the DM reference sequence. The second sheet highlights the non-synonymous SNPs.

Supplementary Data 3
Description: Phenotype data of the Sli transgenics and controls. For all the transgenic lines and controls, the first sheet lists the fertility data and the following sheet lists the microscopic analysis of pollen-tube growth. Supplementary Data 4 Detailed description of the plant materials used for this study.
