# Supplementary material for: Neofunctionalisation of the Sli gene leads to self-compatibility and facilitates precision breeding in potato
Source: Nat Commun. 2021 Jul 6;12:4141. doi: 10.1038/s41467-021-24267-6 (PMC8260583; doi:10.1038/s41467-021-24267-6)
Supplement: Supplementary file 7 — Reporting Summary [file 41467_2021_24267_MOESM7_ESM.pdf]

nature research

Corresponding author(s): Dr, Christian W.B. Bachem

Last updated by author(s): 2021/06/3

# Reporting Summary

Nature Research wishes to improve the reproducibility of the work that we publish. This form provides structure for consistency and transparency in reporting. For further information on Nature Research policies, see our Editorial Policies and the Editorial Policy Checklist.

## Statistics

For all statistical analyses, confirm that the following items are present in the figure legend, table legend, main text, or Methods section.

| n/a | Confirmed | |
|---|---|---|
| ☐ | ☒ | The exact sample size ($n$) for each experimental group/condition, given as a discrete number and unit of measurement |
| ☐ | ☒ | A statement on whether measurements were taken from distinct samples or whether the same sample was measured repeatedly |
| ☒ | ☐ | The statistical test(s) used AND whether they are one- or two-sided<br>*Only common tests should be described solely by name; describe more complex techniques in the Methods section.* |
| ☒ | ☐ | A description of all covariates tested |
| ☒ | ☐ | A description of any assumptions or corrections, such as tests of normality and adjustment for multiple comparisons |
| ☐ | ☒ | A full description of the statistical parameters including central tendency (e.g. means) or other basic estimates (e.g. regression coefficient) AND variation (e.g. standard deviation) or associated estimates of uncertainty (e.g. confidence intervals) |
| ☒ | ☐ | For null hypothesis testing, the test statistic (e.g. $F$, $t$, $r$) with confidence intervals, effect sizes, degrees of freedom and $P$ value noted<br>*Give P values as exact values whenever suitable.* |
| ☒ | ☐ | For Bayesian analysis, information on the choice of priors and Markov chain Monte Carlo settings |
| ☒ | ☐ | For hierarchical and complex designs, identification of the appropriate level for tests and full reporting of outcomes |
| ☒ | ☐ | Estimates of effect sizes (e.g. Cohen's $d$, Pearson's $r$), indicating how they were calculated |

*Our web collection on statistics for biologists contains articles on many of the points above.*

## Software and code

Policy information about availability of computer code

| | |
|---|---|
| Data collection | No specialised software was used during data collection |
| Data analysis | For mapping the Sli locus, MapQTL 5 and Join Map 4.1 software were used (Kyazma, Wageningen, NL). For bioinformatic analysis we used Hiset2 v. 2.1.0 and StringTie v.2.1.1. In addition, general purpose software such as Microsoft Excel and RStudio 1.2.5033 were used for statistical analysis. |

For manuscripts utilizing custom algorithms or software that are central to the research but not yet described in published literature, software must be made available to editors and reviewers. We strongly encourage code deposition in a community repository (e.g. GitHub). See the Nature Research guidelines for submitting code & software for further information.

## Data

Policy information about availability of data

All manuscripts must include a data availability statement. This statement should provide the following information, where applicable:

- Accession codes, unique identifiers, or web links for publicly available datasets
- A list of figures that have associated raw data
- A description of any restrictions on data availability

The Solyntus genome sequence and raw sequence reads are available on NCBI under accession PRJNA631911 [https://www.ncbi.nlm.nih.gov/sra/?term=PRJNA631911]. The Solyntus genome assembly and annotation files are available from WUR [https:// www.plantbreeding.wur.nl/Solyntus/].
The RNA sequencing data from germinated pollen is available on the NCBI short read archive under accession PRJNA713577 [https://www.ncbi.nlm.nih.gov/sra/?term=PRJNA713577].
Other data are available in the source data file or will be made available upon request.

# Field-specific reporting

Please select the one below that is the best fit for your research. If you are not sure, read the appropriate sections before making your selection.

☒ Life sciences ☐ Behavioural & social sciences ☐ Ecological, evolutionary & environmental sciences

For a reference copy of the document with all sections, see nature.com/documents/nr-reporting-summary-flat.pdf

# Life sciences study design

All studies must disclose on these points even when the disclosure is negative.

| | |
|---|---|
| Sample size | For molecular analysis (such as qRT/PCR), at least three independent replicates were used. For cytological analysis (such as styles at least five samples were analyzed from each genotype. |
| Data exclusions | No data was excluded |
| Replication | For mapping populations, the genotypes were phenotyped once. For the recombinant screening, two clones of each genotype were phenotyped. For the transgenic plants, 3-6 independent clones were phenotyped in two independent experiments. |
| Randomization | Bandomization was not applicable in the experiments performed |
| Blinding | No prior knowledge of experimental outcomes was available that would bias the results and thus blinding was unnecessary in the described experiments |

# Reporting for specific materials, systems and methods

We require information from authors about some types of materials, experimental systems and methods used in many studies. Here, indicate whether each material, system or method listed is relevant to your study. If you are not sure if a list item applies to your research, read the appropriate section before selecting a response.

## Materials & experimental systems

| n/a | Involved in the study | |
|---|---|---|
| ☒ | ☐ | Antibodies |
| ☒ | ☐ | Eukaryotic cell lines |
| ☒ | ☐ | Palaeontology and archaeology |
| ☒ | ☐ | Animals and other organisms |
| ☒ | ☐ | Human research participants |
| ☒ | ☐ | Clinical data |
| ☒ | ☐ | Dual use research of concern |

## Methods

| n/a | Involved in the study | |
|---|---|---|
| ☒ | ☐ | ChIP-seq |
| ☒ | ☐ | Flow cytometry |
| ☒ | ☐ | MRI-based neuroimaging |
